# VEGF165-induced vascular permeability requires NRP1 for ABL-mediated SRC family kinase activation

**DOI:** 10.1084/jem.20160311

**Published:** 2017-04-03

**Authors:** Alessandro Fantin, Anastasia Lampropoulou, Valentina Senatore, James T. Brash, Claudia Prahst, Clemens A. Lange, Sidath E. Liyanage, Claudio Raimondi, James W. Bainbridge, Hellmut G. Augustin, Christiana Ruhrberg

**Affiliations:** 1 UCL Institute of Ophthalmology, University College London, London EC1V 9EL, England, UK; 2 Yale Cardiovascular Research Center, New Haven, CT 06511; 3 Division of Vascular Oncology and Metastasis, German Cancer Research Center (DKFZ-ZMBH Alliance), 69120 Heidelberg, Germany; 4 Department of Vascular Biology and Tumor Angiogenesis (CBTM), Medical Faculty Mannheim, Heidelberg University, 68167 Mannheim, Germany

## Abstract

The vascular endothelial growth factor (VEGF) isoform VEGF165 stimulates vascular growth and hyperpermeability. Whereas blood vessel growth is essential to sustain organ health, chronic hyperpermeability causes damaging tissue edema. By combining in vivo and tissue culture models, we show here that VEGF165-induced vascular leakage requires both VEGFR2 and NRP1, including the VEGF164-binding site of NRP1 and the NRP1 cytoplasmic domain (NCD), but not the known NCD interactor GIPC1. In the VEGF165-bound receptor complex, the NCD promotes ABL kinase activation, which in turn is required to activate VEGFR2-recruited SRC family kinases (SFKs). These results elucidate the receptor complex and signaling hierarchy of downstream kinases that transduce the permeability response to VEGF165. In a mouse model with choroidal neovascularisation akin to age-related macular degeneration, NCD loss attenuated vessel leakage without affecting neovascularisation. These findings raise the possibility that targeting NRP1 or its NCD interactors may be a useful therapeutic strategy in neovascular disease to reduce VEGF165-induced edema without compromising vessel growth.

## Abstract (precis)

Fantin et al. show that the VEGF isoform VEGF165 signals through a complex of VEGFR2 and NRP1, in which the NRP1 cytoplasmic domain promotes the ABL-mediated activation of SRC family kinases to evoke a hyperpermeability response, a known cause of pathological edema.

## Introduction

Diseases characterized by ischemia affecting the brain, retina, heart, and limb significantly impact human health, and the therapeutic induction of blood vessel growth by delivery of the vascular endothelial growth factor (VEGF) has the potential to alleviate tissue ischemia (Potente et al., 2011). However, VEGF also increases vascular hyperpermeability, both acutely at injury sites and over prolonged periods in chronic conditions with associated edema; for example, in neovascular eye disease, pulmonary vascular disease, and cancer (Ma et al., 2012; Greenberg and Jin, 2013; Barratt et al., 2014). To date, a poor understanding of the molecular mechanisms that distinguish VEGF-mediated permeability from other VEGF responses has hampered the design of therapies that selectively target VEGF-induced vessel leak and therefore edema.

The tyrosine kinase receptor VEGFR2 has been implicated as the main VEGF receptor in endothelial permeability signaling in various organs, including the lung, skin, and brain (Murohara et al., 1998; Weis et al., 2004; Weis and Cheresh, 2005; Sun et al., 2012; Hudson et al., 2014; Li et al., 2016). In response to VEGF, VEGFR2 activates SRC family kinases (SFKs) and the ABL kinases ABL1 and ABL2 (also known as ARG) to mediate VEGF-induced vascular permeability (Eliceiri et al., 1999; Aman et al., 2012; Anselmi et al., 2012; Sun et al., 2012; Chislock and Pendergast, 2013). However, a VEGF mutant with low VEGFR2 affinity retains the ability to evoke intradermal vascular hyperpermeability (Stacker et al., 1999), raising the possibility that VEGFR2 either recruits a VEGF-binding co-receptor or that VEGF can engage an alternative receptor for permeability signaling. In humans, VEGF is made as three main isoforms termed VEGF121, VEGF165, and VEGF189, with VEGF165 considered the most pathological VEGF isoform (Usui et al., 2004). In addition to having a strong affinity for extracellular matrix, VEGF165 also differs from VEGF121 by its ability to bind neuropilin 1 (NRP1), a noncatalytic co-receptor that forms VEGF165-dependent complexes with VEGFR2 in endothelial cells (ECs; Soker et al., 1998). Complexes are then trafficked into signaling endosomes, thereby protecting VEGFR2 from premature dephosphorylation and enabling sustained activation of the ERK1 and ERK2 kinases for arteriogenesis (Lanahan et al., 2013).

NRP1 has also been implicated in vascular permeability signaling (Raimondi et al., 2016). Intradermal vascular leakage induced by VEGF164, the murine equivalent of VEGF165, is defective in mice lacking endothelial NRP1 expression, even though they retain VEGFR2 (Acevedo et al., 2008). Agreeing with an important role for NRP1 in VEGF164-induced vascular permeability, a peptide blocking VEGF164 binding to NRP1 inhibits serum albumin leak in a mouse model of diabetic retinal injury (Wang et al., 2015), and function-blocking antibodies for NRP1 suppress intradermal vascular leak induced by VEGF164 injection (Teesalu et al., 2009), as well as VEGF164-induced pulmonary vascular leak (Becker et al., 2005). However, other studies have argued against an important role for NRP1 in VEGF-induced vascular permeability, with one study showing that an antibody blocking VEGF164 binding to NRP1 impaired corneal neovascularisation, but not VEGF164-induced intradermal vascular permeability in mice (Pan et al., 2007), and another study finding that NRP1 deletion does not impair VEGF164-induced permeability of retinal vasculature (Cerani et al., 2013). Additionally, C-end-Rule peptides, which bind NRP1, can induce permeability independently of VEGFR2 activation (Roth et al., 2016). The relative importance of VEGFR2 and NRP1 for VEGF-induced vascular permeability signaling has therefore remained unclear. Moreover, it is not known how NRP1 function may intersect with ABL kinase or SFK activation and whether these downstream kinases operate in a regulatory hierarchy to convey permeability signals.

Here, we have compared VEGF164-induced intradermal vascular leakage in a comprehensive range of mouse mutants to conclusively demonstrate an absolute requirement for VEGFR2 and a strong dependency on NRP1, including its VEGF164-binding pocket and the NRP1 cytoplasmic domain (NCD). We further show that endothelial NRP1 and the NCD are required for VEGF165-induced SFK phosphorylation, which also depends on the VEGFR2-dependent activation of ABL kinases upstream of SFK activation. Moreover, in a mouse model of VEGF-dependent neovascular pathology akin to exudative age-related macular degeneration, NCD-deficient mice had significantly reduced ocular vascular leakage, but neovascularisation was unchanged. Together, our findings suggest that targeting the NCD-mediated signaling pathway may provide a novel therapeutic strategy to selectively treat VEGF165-induced vascular leak without compromising other VEGF functions.

## Results

### VEGF164-induced vascular leakage depends on VEGFR2, NRP1, and VEGF-binding to NRP1

**Figure 1.**
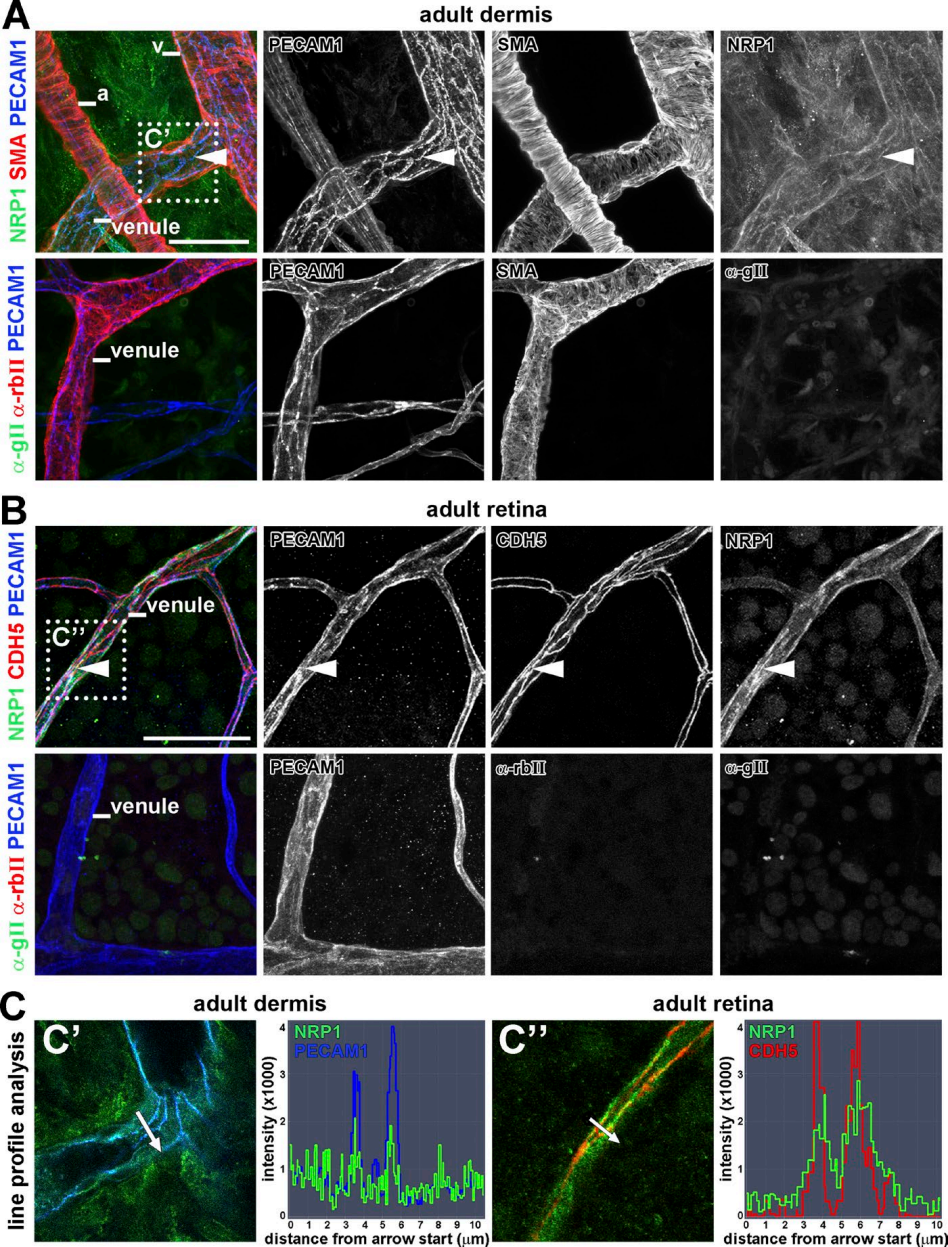
**NRP1 localization in adult vascular endothelium.** Whole-mount immunostaining of adult mouse ear dermis for NRP1, PECAM1, and SMA (A) and adult mouse retina for NRP1, PECAM1, and CDH5 (B), including control staining for the secondary antibodies used to detect NRP1 (anti–goat) and CDH5 (anti–rabbit), together with the primary and secondary antibody for PECAM1 (three independent experiments). Arrowheads indicate examples of endothelial junctions sites enriched for NRP1 in venules. a, artery; v, vein. (C) Single optical sections from the boxed areas in A and C’ and B and C’’ were analyzed for pixel intensity along a virtual line crossing the blood vessel. Bars, 50 µm.

NRP1 is expressed in developing and pathological blood vessels to promote angiogenesis (Fantin et al., 2013; Raimondi et al., 2014; Aspalter et al., 2015). To determine whether NRP1 expression is maintained in quiescent endothelium, we performed whole mount immunolabeling of adult mouse dermis and retina with a previously validated antibody for NRP1 (Fantin et al., 2010). In both tissues, NRP1 localized to PECAM1-positive capillaries, arteries, and veins, including venules (Fig. 1, A and B), consistent with a role for NRP1 in regulating vascular permeability. Moreover, NRP1 in dermal (Fig. 1, A and C’) and retinal (Fig. 1, B and C’’) venules appeared to be concentrated in areas enriched for the adherens junction proteins PECAM1 and CDH5 (VE-cadherin).

**Figure 2.**
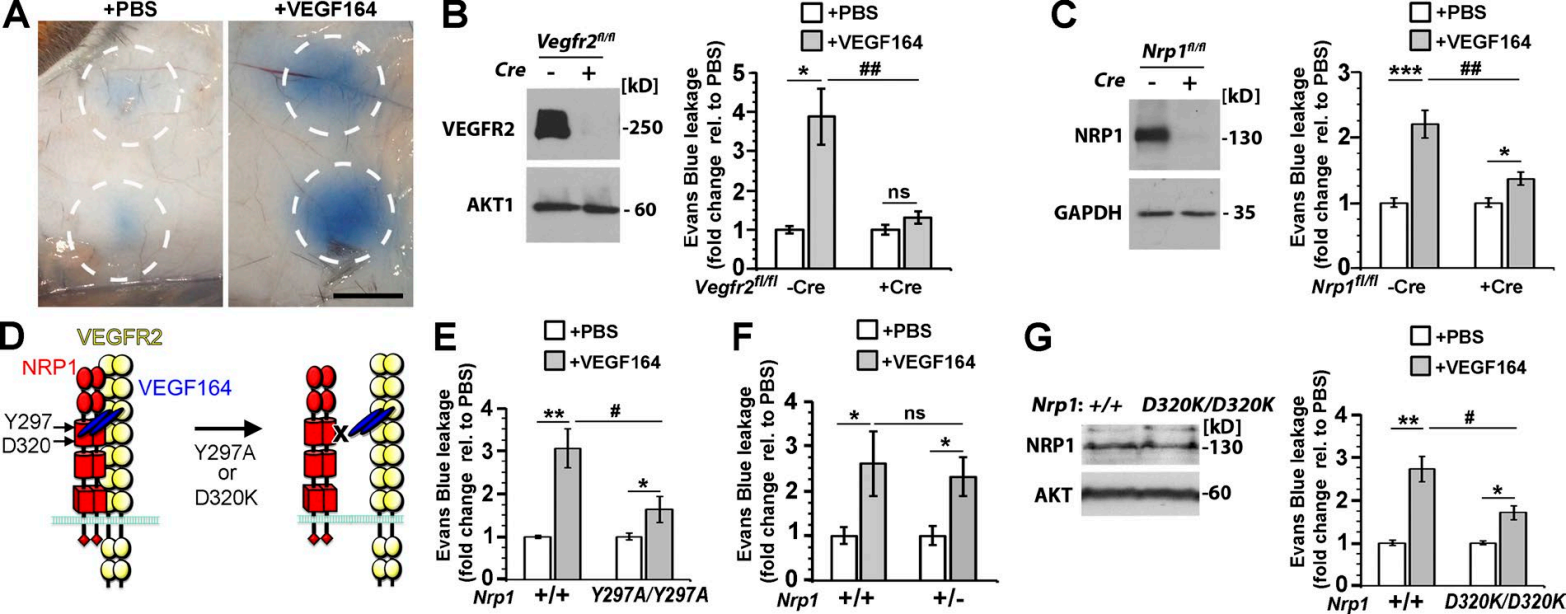
**VEGF164-induced vascular leakage depends on VEGFR2, NRP1 and VEGF-binding to NRP1.** (A) Evans Blue leaks from the circulation into the dermis after intradermal injection of VEGF164, but not PBS; the circles indicate the tissue area around the injection sites that was excised for dye extraction. Bar, 1 cm. (B and C) *Vegfr2^fl/fl^* (B) and *Nrp1^fl/fl^* (C) mice expressing or lacking the endothelial *Cdh5-CreERT2* transgene were tamoxifen treated to induce gene deletion; immunoblotting of liver (B) or skin (C) lysates with the indicated antibodies (left) confirmed gene deletion, whereas Miles assays with PBS versus VEGF164 (right) showed defective VEGF164-induced leakage. (D) Schematic representation of NRP1 mutants with defective VEGF164 binding to NRP1. (E–G) Miles assay with PBS versus VEGF164 in mutant and wild-type littermates of the indicated genotypes. Immunoblotting of skin lysates with the indicated antibodies (G) showed normal NRP1 levels in *Nrp1^D320K/D320K^* mice compared with littermate controls. In B–G, leakage was measured as optical density and expressed as fold change relative to PBS, mean ± SEM; *n* = 5 each (B and G), *n* = 5 controls, *n* = 6 mutants (C), *n* = 8 controls, *n* = 10 mutants (E), *n* = 4 controls, *n* = 7 mutants (F); asterisks indicate significant P-values for permeability-inducing agents versus PBS: *, P < 0.05; **, P < 0.01; ***, P < 0.001; ns, not significant, P > 0.05; paired Student’s *t* test. Hash tags indicate significant P-values for permeability-induction in mutants versus controls (^#^, P < 0.05; ^##^, P < 0.01; ns, P > 0.05; unpaired Student’s *t* test).

To directly compare the genetic requirement of NRP1 and VEGFR2 for VEGF164-induced vascular permeability, we used the Miles assay, which measures the extravasation of Evans blue–labeled serum albumin after intradermal injection of permeability-enhancing agents (Miles and Miles, 1952; Senger et al., 1983; Brkovic and Sirois, 2007). In this assay, VEGF induces vascular permeability independently of its effect on systemic blood pressure (Li et al., 2016). As previously shown (Aman et al., 2012; Sun et al., 2012; Li et al., 2016), wild-type mice exhibited prominent dye leakage 20 min after injection of VEGF164, but little or no dye leakage after PBS injection (Fig. 2 A). In contrast to their control littermates, endothelial *Vegfr2*-null mice did not respond to VEGF164 injection with increased vessel leakage (Fig. 2 B). Endothelial *Nrp1*-null mice also showed a significant reduction in VEGF164-induced leakage compared with littermate controls, but, unlike endothelial *Vegfr2*-null mice, had a residual response (Fig. 2 C). Both NRP1 and VEGFR2 are therefore essential for VEGF164-induced vascular permeability, with VEGFR2 being absolutely required and NRP1 making an indispensable contribution for a robust response.

We next examined whether VEGF164 binding to NRP1 is required for permeability induction. For these experiments, we performed Miles assays with two strains of mice lacking VEGF164 binding to NRP1 (Fig. 2 D), i.e., mice with homozygous Y297A or D320K mutations, as previously described (Fantin et al., 2014; Gelfand et al., 2014). *Nrp1^Y297A/Y297A^* mice had significantly impaired VEGF164-induced permeability (Fig. 2 E). As these mice have reduced NRP1 levels in addition to defective VEGF164 binding (Fantin et al., 2014), we also examined *Nrp1^+/−^* mice with reduced NRP1 levels (Fantin et al., 2015). Unlike Nrp1Y297A/Y297A mice, *Nrp1^+/−^* mice showed similar VEGF164-induced leakage as littermate wild-type controls (Fig. 2 F). Moreover, *Nrp1^D320K/D320K^* mice with normal NRP1 levels (Fig. 2 G) also had significantly reduced VEGF164-induced permeability (Fig. 2 G).

Together, our findings are compatible with a model in which VEGF164 binding to NRP1 induces complex formation between NRP1 and VEGFR2 (Soker et al., 2002) to create an obligate holoreceptor in which VEGFR2 is required, but depends on NRP1 to evoke a maximal permeability response to VEGF164.

### VEGF164-induced vascular leakage depends on the NCD, but not GIPC1

**Figure 3.**
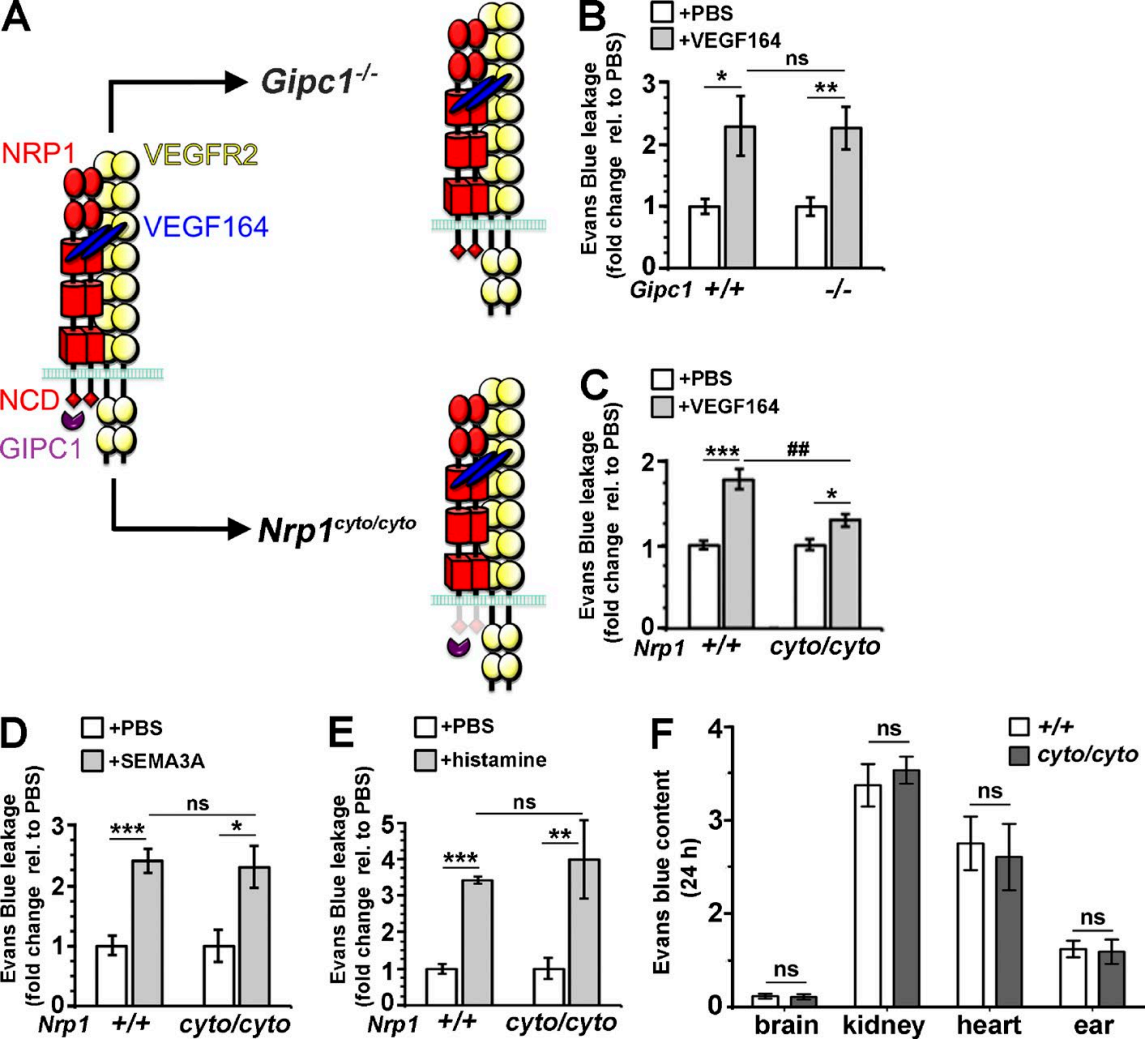
**The NCD is required to mediate VEGF164-induced vascular leakage, but does not regulate baseline vascular permeability.** (A) Schematic representation of mutations that impair NRP1 intracellular activity. (B–E) Miles assay with the indicated substances in mutant and wild-type littermates of the indicated genotypes; leakage was measured as optical density and expressed as fold change relative to PBS, mean ± SEM; *n* = 6 controls, *n* = 5 mutants (B), *n* = 7 each (C), *n* = 5 each (D), *n* = 4 each (E); asterisks indicate significant P-values for permeability-inducing agents versus PBS (*, P < 0.05; **, P < 0.01; ***, P < 0.001; paired Student’s *t* test), and hash tags indicate significant P-values for permeability-induction in mutants versus controls (^##^, P < 0.01; ns, not significant, P > 0.05; unpaired Student's *t* test). (F) Evans Blue content in the indicated organs 24 h after systemic injection in *Nrp1^cyto/cyto^* mice and wild-type littermates. Values are normalized to tissue weight and Evans Blue levels in the blood; mean ± SEM; *n* = 3 each; ns, P > 0.05; unpaired Student’s *t* test.

As GIPC1 (synectin) promotes complex formation between NRP1 and VEGFR2 (Prahst et al., 2008), and NRP1 promotes VEGF164-induced arteriogenesis by recruiting GIPC1 to the NCD (Lanahan et al., 2013), we next asked whether GIPC1 binding to the NCD is also required for VEGF164-induced vascular leakage (Fig. 3 A). However, mice lacking GIPC1 (Chittenden et al., 2006) showed similar VEGF164-induced vascular leakage compared with their control littermates (Fig. 3 B). In contrast, *Nrp1^cyto/cyto^* mice expressing a mutant form of NRP1 lacking the NCD (Fantin et al., 2011) showed significantly reduced VEGF164-induced dye leakage compared with controls (Fig. 3 C), similar to endothelial *Nrp1*-null, *Nrp1^Y297A/Y297A^*, and *Nrp1^D320K/D320K^* mutants. The finding that NRP1 promotes vascular leakage through the NCD independently of GIPC1 distinguishes permeability from arteriogenic VEGF signaling, despite a shared dependence of both responses on the NCD.

### The NCD is dispensable for SEMA3A- and histamine-induced vascular permeability

As SEMA3A signals through NRP1 to induce vascular leakage in the skin and retina (Acevedo et al., 2008; Cerani et al., 2013), we next examined whether SEMA3A-induced vascular permeability was also NCD-dependent. However, dye extravasation in response to SEMA3A was similar in *Nrp1^cyto/cyto^* and wild-type littermates (Fig. 3 D). These observations are consistent with prior findings in the nervous system, where SEMA3A binds NRP1, but recruits a plexin co-receptor that can transduce signals independently of the NCD (Epstein et al., 2015). Histamine acts via G-protein coupled receptors to induce vascular hyperpermeability in many inflammatory settings (Miles and Miles, 1952). Similar to SEMA3A, histamine increased dye leakage similarly in *Nrp1^cyto/cyto^* and wild-type mice (Fig. 3 E). The NCD is therefore specifically important for vascular hyperpermeability in response to VEGF164.

### The NCD is not required to maintain the baseline vascular barrier to serum leak

To determine whether NCD loss alters baseline vascular permeability to serum proteins, we injected Evans Blue into the circulation of *Nrp1^cyto/cyto^* mice and wild-type littermate controls. After 24 h, dye extravasation was low in the brain due to the tight blood–brain–barrier, high in the kidney with its endothelial fenestrations and intermediate in organs such as the heart and the ear skin, with similar dye extravasation in both genotypes (Fig. 3 F). Together with our observations in the Miles assay, these findings suggest that NCD loss does not obviously alter the endothelial barrier to serum proteins under physiological circumstances, but is selectively required for the acute hyperpermeability response to VEGF164.

### NRP1 promotes VEGF165-induced SFK activation

**Figure 4.**
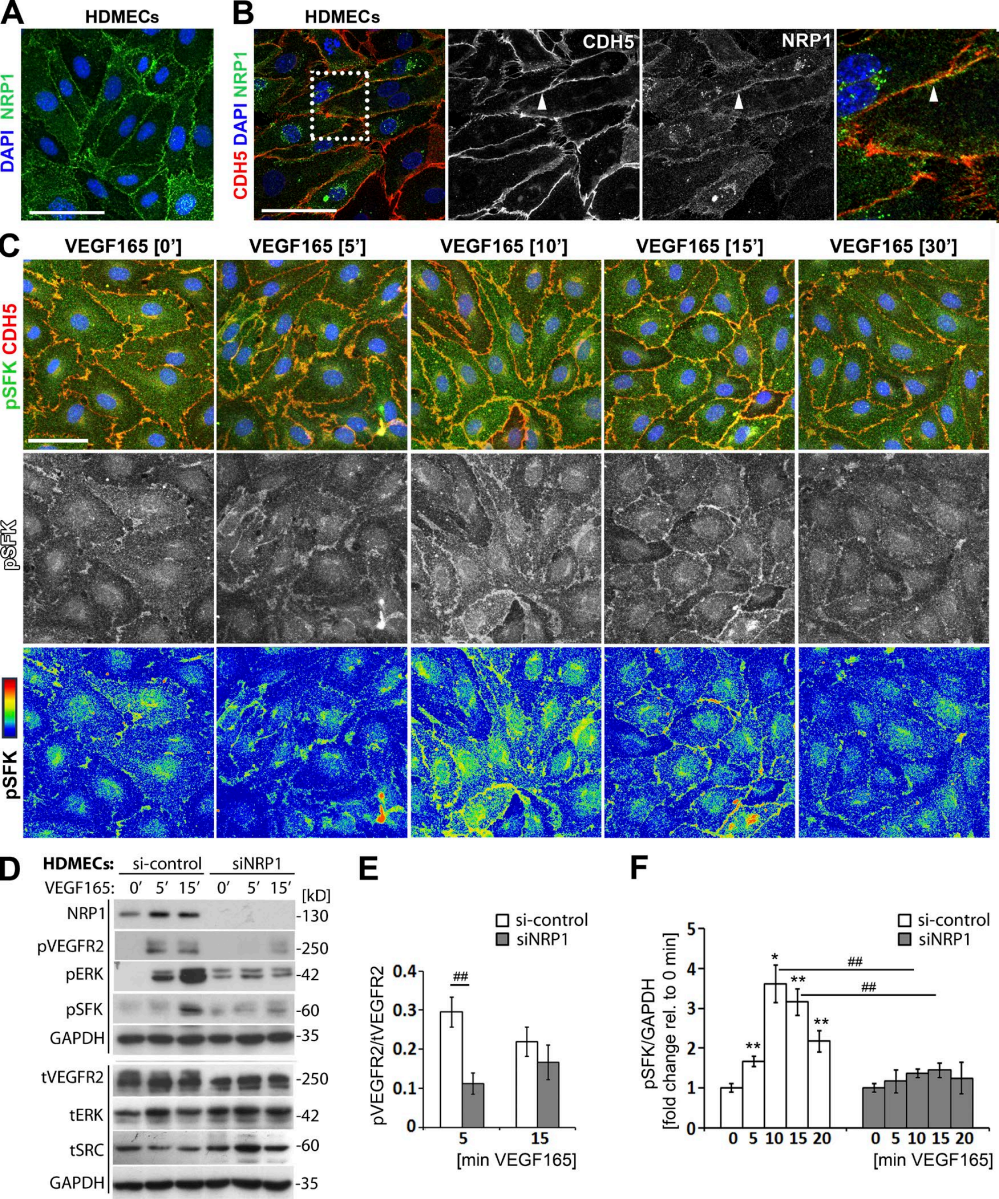
**NRP1 loss impairs VEGF165-induced SFK activation in human ECs.** (A and B) Immunostaining of confluent HDMEC cultures in growth medium under nonpermeabilizing conditions with an antibody specific for human NRP1 (A) or under permeabilizing conditions with antibodies for CDH5 and NRP1 together with DAPI to visualize cell nuclei (B); three independent experiments. Single channels in B are shown separately in grayscale, and the boxed area is shown in higher magnification on the right. Bar, 50 µm. (C) Immunostaining of confluent HDMEC cultures under permeabilizing conditions for pSFK together with CDH5 and DAPI after serum withdrawal, followed by stimulation with VEGF165 for the indicated times (three independent experiments). The corresponding single pSFK channels are shown beneath each panel in grayscale as well as the rainbow pixel intensity scale. Bar, 50 µm. (D–F) Confluent HDMEC cultures transfected with si-control or siNRP1 were serum-starved and treated with VEGF165 for the indicated times. Lysates were used for immunoblotting with the indicated antibodies (D), followed by quantification of pVEGFR2 (Y1175; E) and pSFK (F) induction relative to tVEGFR2 and GAPDH, respectively. Each of the two vertical lines indicates a group of immunoblots from a single gel, with both gels containing aliquots of the same protein lysate. Data for si-control and siNRP1-treated cells are expressed as ratio (E) or fold change, for VEGF165 treatment at different time points relative to 0 min (F) mean ± SEM; *n* = 4 independent experiments; asterisks indicate significant P-values for pSFK induction after VEGF165 treatment (*, P < 0.05; **, P < 0.01; paired Student’s *t* test). Hash tags indicate significant P-values for reduced pVEGFR2 and pSFK levels in siNRP1 versus si-control at the corresponding time points (^##^, P < 0.01; unpaired Student’s *t* test).

Two SFK members, SRC and YES1, are tyrosine phosphorylated to transduce signals important for VEGF165-induced vascular permeability (Eliceiri et al., 1999; Scheppke et al., 2008). We therefore examined the requirement of NRP1 for VEGF165-induced SFK activation in human dermal microvascular ECs (HDMECs), which are known to form monolayers with relatively well-organized intercellular contacts (Kluger et al., 2013). Immunostaining of confluent, nonpermeabilized HDMECs in normal growth conditions showed NRP1 localization on the cell surface, including areas of cell–cell contact (Fig. 4 A). Immunostaining for NRP1 and an intracellular epitope of CDH5 after permeabilization confirmed localization of a NRP1 subset to areas of cell–cell contact in HDMECs (Fig. 4 B), similar to the pattern observed in skin and retinal vasculature (Fig. 1, A and B). We next performed immunostaining with an antibody raised against the phosphorylated tyrosine (Y) 419 of activated SRC that also recognizes the phosphorylated forms of other SFKs due to high sequence conservation around the phosphosite. We observed that levels of phosphorylated SFKs (pSFK) in HDMECs peaked 10 and 15 min after VEGF165 stimulation, with an enrichment of pSFK at CDH5-positive junctions (Fig. 4 C). Accordingly, HDMEC monolayers represent a suitable model to investigate NRP1-mediated permeability signaling.

To examine the requirement of NRP1 for VEGF165-mediated pSFK induction, we transfected HDMECs with a previously validated small interference (si) RNA that targets NRP1 or a control nonsense siRNA (Raimondi et al., 2014; Fantin et al., 2015). Immunoblotting validated NRP1 knockdown efficiency and reduced phosphorylation of the VEGFR2 Y1175 (pVEGFR2) and the ERK1/2 T202/Y204 (pERK) residues after VEGF165 stimulation in NRP1-deficient compared with NRP1-expressing cells (Fig. 4, D and E), as previously attributed to impaired VEGFR2 trafficking (Lanahan et al., 2013; Raimondi et al., 2014). Immunoblotting further showed that VEGF165 stimulation increased pSFK levels in control HDMECs, but that this response was attenuated in HDMECs lacking NRP1 (Fig. 4 D). As total SRC levels were increased in NRP1-deficient cells (Fig. 4 D), reduced pSFK activation was not explained by reduced SRC expression. Quantification demonstrated a significant reduction in pSFK activation 10 and 15 min after VEGF165 stimulation in HDMECs lacking NRP1 compared with controls (Fig. 4 F; we normalized pSFK to GAPDH rather than an individual SFK, because the pSFK antibody recognizes the phosphorylated forms of several SFKs). Together, these findings suggest that endothelial NRP1 is essential for VEGF165-induced SFK activation.

### VEGF165-induced SFK activation relies on VEGFR2- and NRP1-mediated ABL kinase activation

**Figure 5.**
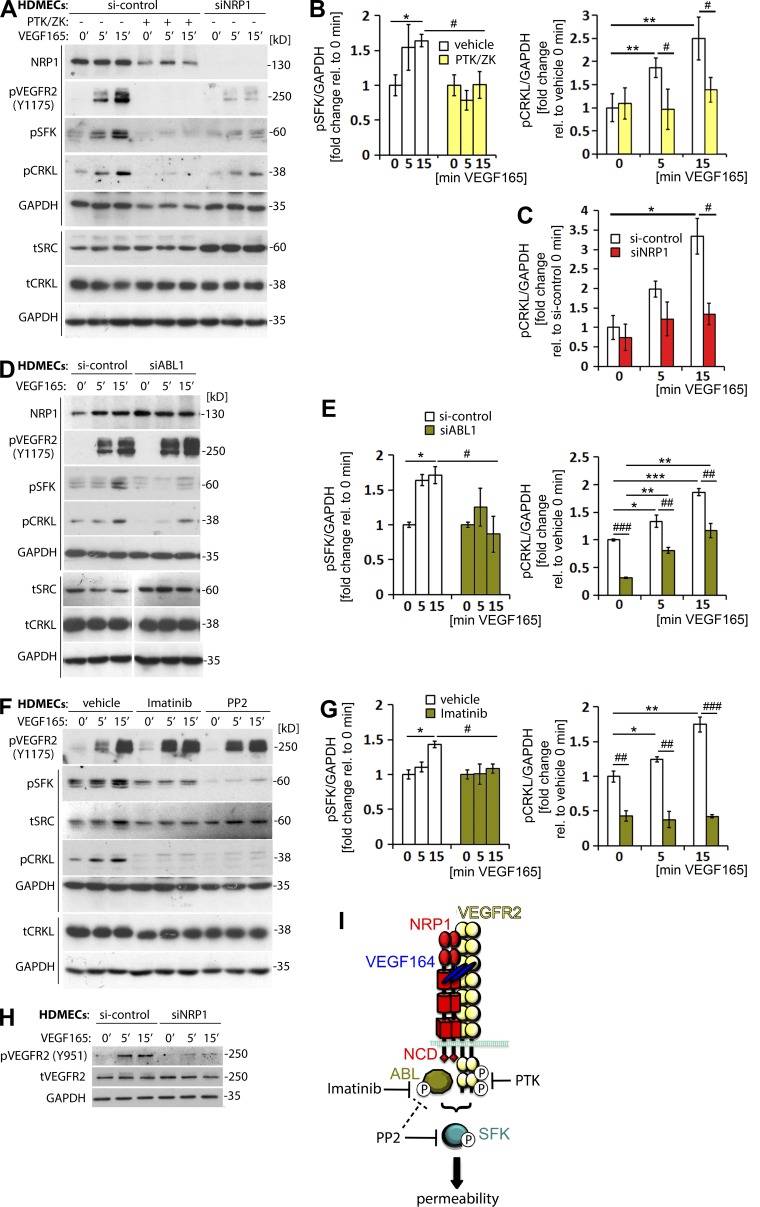
**VEGFR2 and NRP1 are required for VEGF165-induced SFK activation via ABL1.** (A–C) Confluent HDMEC cultures transfected with si-control or siNRP1 were serum-starved and treated with VEGF165 for the indicated times. Cultures were also treated with vehicle (-) or PTK/ZK (+) for 30 min before VEGF165 stimulation. Lysates were used for immunoblotting with the indicated antibodies (A), followed by quantification of pSFK levels (B, left) and pCRKL levels (B’, right, and C) relative to GAPDH (four independent experiments). Each of the two vertical lines indicated a group of immunoblots from a single gel, with both gels containing aliquots of the same protein lysate. (D–E) Confluent HDMEC cultures transfected with si-control or siABL1 were serum-starved and treated with VEGF165 for the indicated times. Lysates were used for immunoblotting with the indicated antibodies (D), followed by quantification of pSFK levels (E, left) and pCRKL levels (E’, right) relative to GAPDH (four independent experiments). Each of the two vertical lines indicates a group of immunoblots from a single gel, with both gels containing aliquots of the same protein lysate. The spacer line (D, bottom) separates lanes 4–6 (left) from lanes 1–3 (right) of immunoblots from the gel in Fig. S1. (F–G) Confluent HDMEC cultures were serum-starved and treated with vehicle, Imatinib or PP2 for 30 min before VEGF165 stimulation for the indicated times. Lysates were used for immunoblotting with the indicated antibodies (F), followed by quantification of pSFK levels (G, left) and pCRKL levels (G’, right) relative to GAPDH (three independent experiments). Each of the two vertical lines indicates a group of immunoblots obtained from a single gel, with both gels containing aliquots of the same protein lysate. In B, E, and G (left) data are expressed as fold change, mean ± SEM, in VEGF165-treated cells at 5 and 15 min relative to 0 min; in C and B, E, and G (right), data are expressed as fold change, mean ± SEM, in VEGF165-treated cells at 5 and 15 min relative to control cells at 0 min; asterisks indicate P-values for induction after VEGF165 treatment (*, P < 0.05; **, P < 0.01; ***, P < 0.001; paired Student’s *t* test); hash tags indicate significant P-values for different treatments at corresponding time points (^#^, P < 0.05; ^##^, P < 0.01; ^###^, P < 0.001; unpaired Student’s *t* test; *n* ≥ 3 independent experiments). (H) Confluent HDMEC cultures transfected with si-control or siNRP1 and serum-starved were treated with VEGF165 for the indicated times and lysates used for immunoblotting with the indicated antibodies (three independent experiments). (I) Model for VEGF165-induced vascular permeability signaling including the point of interference by pharmacological inhibitors used in this study.

VEGF165 stimulation activates ABL1 and ABL2 in human ECs in vitro, and ABL kinase activation is essential for VEGF164-induced vascular permeability in the Miles assay (Aman et al., 2012; Anselmi et al., 2012; Chislock and Pendergast, 2013). However, it has not previously been examined whether VEGFR2 or NRP1 contribute to SFK activation in an ABL kinase-dependent manner. We therefore determined the regulatory hierarchy of these signaling molecules in VEGF165-stimulated HDMEC monolayers. First, we investigated whether VEGFR2 activation is required for ABL or SFK activation by treating HDMECs with PTK/ZK (Vatalanib), a highly specific VEGFR2 inhibitor that abolishes VEGFR2 downstream signaling (Wood et al., 2000), but does not directly target SRC or ABL kinases (VEGFR2 k_d_ 62 nM; SRC, YES1, ABL1, or ABL2 k_d_ not detected under normal assay conditions, i.e., >10 µM; Wodicka et al., 2010; Davis et al., 2011). Immunoblotting confirmed that PTK/ZK impaired VEGF165-induced VEGFR2 activation (Fig. 5 A). PTK/ZK also abrogated pSFK induction (Fig. 5, A and B), consistent with prior work demonstrating a role for VEGFR2 in SRC activation (Sun et al., 2012; Li et al., 2016).

To determine whether VEGFR2 is involved in ABL kinase activation, we examined the VEGF165-induced phosphorylation of CRKL on Y207, an ABL kinase target that is widely used as readout of ABL kinase activation (Sattler and Salgia, 1998). We observed increased pCRKL levels 5 and 15 min after VEGF165 treatment in control, but not PTK/ZK-treated cells (Fig. 5, A and B). The requirement of VEGFR2 for both ABL kinase and SFK activation agrees with our finding that endothelial VEGFR2 is absolutely required for VEGF164-induced vascular permeability in vivo (Fig. 2 B).

NRP1 knockdown also impaired VEGF165-mediated pSFK induction and decreased pCRKL levels (Fig. 5, A and C). In contrast to PTK/ZK treatment, however, NRP1 knockdown reduced pCRKL levels at baseline, i.e., before VEGF165 stimulation (Fig. 5 C). Also different to PTK/ZK treatment, NRP1 knockdown did not prevent the VEGF165-induced pCRKL increase, although pCRKL levels remained significantly lower in NRP1-deficient compared with control cells at all times (Fig. 5 C). Thus, VEGF165-induced ABL kinase activation depends on VEGFR2 completely and on NRP1 partially.

As NRP1 interacts with ABL1 and is required for its activation in fibronectin-stimulated ECs (Raimondi et al., 2014), we next determined whether ABL1 is required for pSFK and pCRKL induction. For this experiment, we transfected HDMEC with previously validated siRNA for ABL1 (Raimondi et al., 2014). Similar to NRP1 knockdown, ABL1 knockdown inhibited pSFK induction after VEGF165 stimulation and decreased overall pCRKL levels, but knockdown did not prevent the VEGF164-induced increase in pCRKL levels (Fig. 5, D and E). The finding that pCRKL levels are similarly reduced and pSFK induction severely compromised in cells lacking NRP1 or ABL1 suggests that NRP1-dependent ABL1 activation is required for pSFK activation, but that NRP1 is not the sole regulator of ABL kinase-dependent pCRKL induction.

We next asked whether ABL2 cooperates with ABL1 to mediate the VEGF164-induced pCRKL induction. For this experiment, we treated HDMECs with Imatinib, which efficiently targets ABL1 and ABL2, but not SRC, YES1, or VEGFR2 (ABL1 k_d_, 1 nM; ABL2 k_d_, 10 nM vs. SRC, YES1, and VEGFR2 k_d_, not detected under normal assay conditions, i.e., >10 µM; ABL1 IC_50_, 0.025–0.2 µM vs. SRC IC_50_ >100 µM; Buchdunger et al., 1996; Deininger et al., 2005; Davis et al., 2011). As expected, Imatinib inhibited pCRKL induction without affecting VEGFR2 phosphorylation (Fig. 5, F and G’). Moreover, and as observed for NRP1 or ABL1 knockdown, Imatinib significantly impaired VEGF165-induced SFK activation despite its poor specificity for SFKs (Fig. 5, F and G). PP2, a dual SFK and ABL kinase inhibitor (Tatton et al., 2003), also impaired pSFK induction and additionally suppressed baseline SFK phosphorylation (Fig. 5 F). In contrast, neither inhibitor impaired VEGFR2 activation (Fig. 5 F). These findings suggest that ABL kinase activity is required for VEGF165-induced SFK activation downstream of VEGFR2.

The VEGF164-induced phosphorylation of the VEGFR2 Y949 residue (Y951 in humans) is essential to recruit SH2D2A (also known as T cell–specific adaptor, TSAd), which then recruits SRC to VEGFR2 for vascular permeability signaling (Sun et al., 2012; Li et al., 2016). Consistent with an important role for NRP1 in VEGF164-induced vascular permeability, we observed that VEGF165-induced VEGFR2 Y951 phosphorylation was reduced in NRP1-deficient HDMECs compared with controls (Fig. 5 H).

Collectively, our findings demonstrate that VEGF165 signals via VEGFR2 in a NRP1 and ABL kinase dependent manner to activate SFKs and increase vascular permeability (Fig. 5 I).

### VEGF164-induced vascular permeability signaling via SFKs relies on the NCD

**Figure 6.**
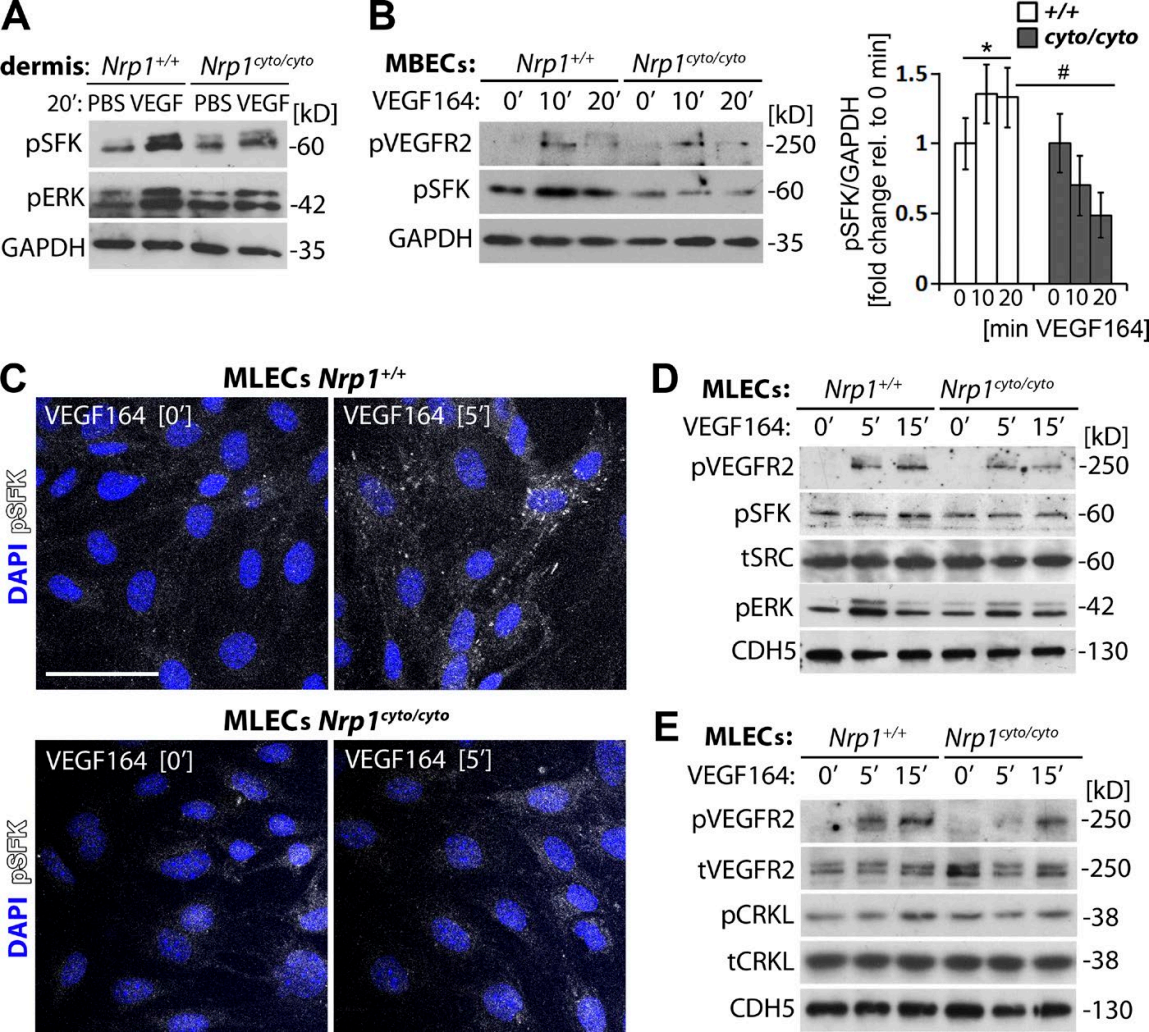
**NCD loss impairs VEGF164-induced SFK activation in the mouse.** (A) *Nrp1^cyto/cyto^* and wild-type ears were injected with VEGF164 or PBS for 20 min and lysates used for immunoblotting with the indicated antibodies (two independent experiments). (B) Confluent MBECs from *Nrp1^cyto/cyto^* and wild-type brains were serum-starved and treated with VEGF164 for the indicated time points. Lysates were used for immunoblotting with the indicated antibodies (left), followed by quantification of pSFK relative to GAPDH levels (right). Data are expressed as fold change, mean ± SEM, in VEGF164-treated cells at 10 and 20 min relative to 0 min; *n* = 3 independent experiments; asterisk indicates significant P-value for induction after VEGF164 treatment (*, P < 0.05; paired Student’s *t* test); hash tag indicates significant P-value for different genotypes at corresponding time point (^#^, P < 0.05; unpaired Student’s *t* test). (C–E) Confluent MLECs from *Nrp1^cyto/cyto^* and wild-type lungs were serum-starved and treated with VEGF164 for the indicated times and immunostained under permeabilizing conditions using an antibody for pSFK (C) or lysed for immunoblotting with the indicated antibodies (D and E); cells were counterstained with DAPI (two independent experiments each). Bar, 50 µm.

We next determined whether the NCD was required for endothelial pSFK induction. After injection of VEGF164 versus PBS into ear dermis, immunoblotting of lysates from tissues surrounding the injection site demonstrated that VEGF164 induced SFK and ERK1/2 phosphorylation in wild-type mice, and that this response was impaired in NCD-deficient mice (Fig. 6 A). We next analyzed confluent, nonpassaged primary mouse brain ECs (MBECs); immunoblotting confirmed that VEGF164 induced VEGFR2 Y1173 phosphorylation (corresponding to Y1175 in human VEGFR2). Moreover, VEGF164-induced SFK phosphorylation in wild-type, but not NCD-deficient MBECs (Fig. 6 B). Immunostaining and immunoblotting of confluent primary mouse lung ECs (MLECs) also confirmed that VEGF164 increased pSFK levels in ECs from wild-type, but not NCD-deficient mice (Fig. 6, C and D). Three different assays therefore showed that NCD loss impairs VEGF164-induced SFK activation. NCD loss also impaired VEGF164-induced CRKL activation in MLECs (Fig. 6 E). Together, these findings suggest that the NCD enables VEGF164-induced ABL kinase and SFK activation in ECs.

### The NCD promotes vascular hyperpermeability, but not angiogenesis in neovascular eye disease

**Figure 7.**
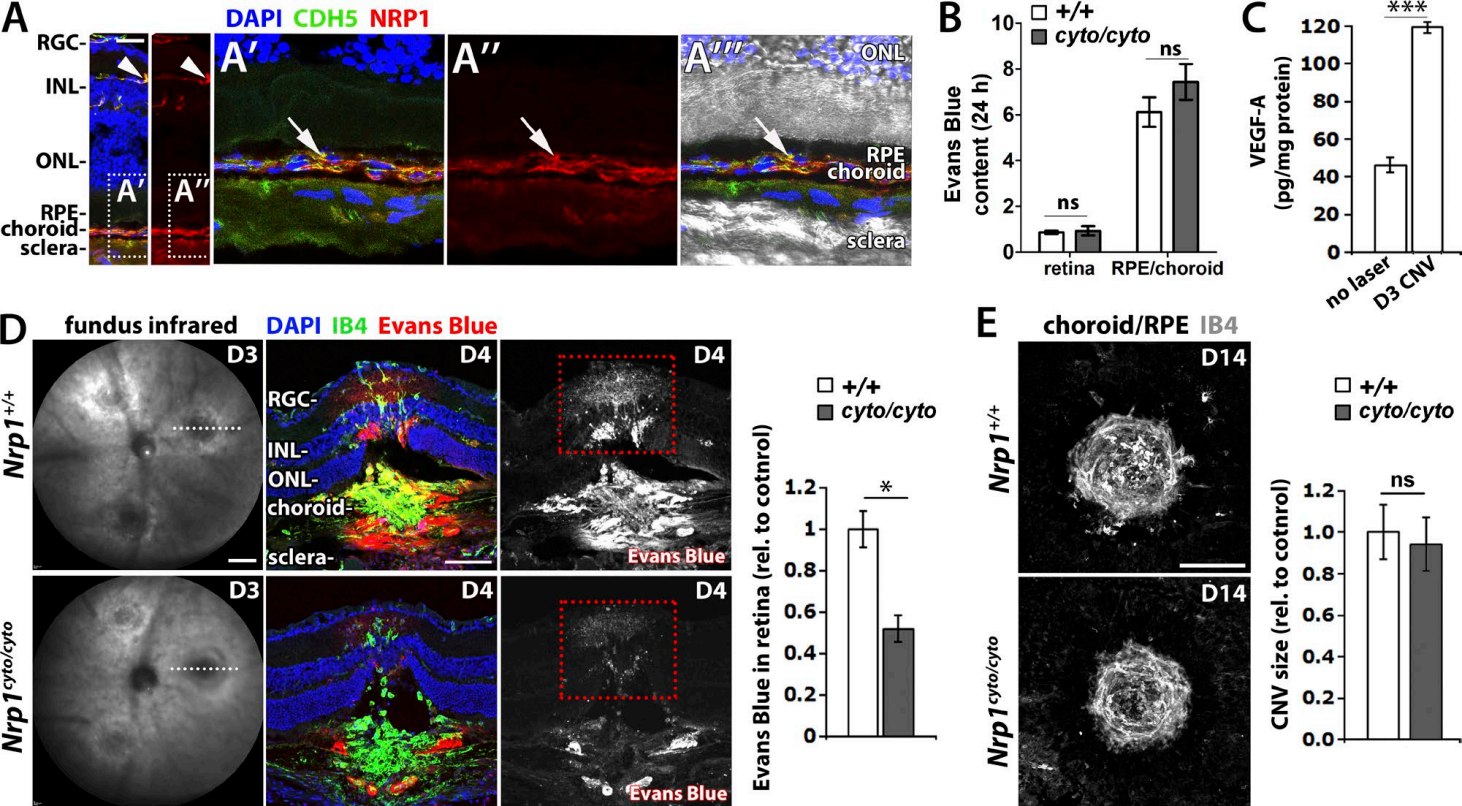
**The NCD promotes vascular leakage, but not neovascularisation, in a mouse model of CNV.** (A) Adult eye sections immunostained for NRP1 and CDH5; nuclei were counterstained with DAPI (two independent experiments); NRP1 staining is shown separately on the right. An extension of the squared area is shown at higher magnification in (A’–A’’’); the NRP1 channel is shown separately in (A’’); the DIC image is superimposed in A’’’. RGC, retinal ganglion cell layer; INL, inner nuclear layer; ONL, outer nuclear layer; RPE, retinal pigment epithelium. (B) Evans Blue content in the indicated ocular tissues in *Nrp1^cyto/cyto^* mice and wild-type littermates; mean ± SEM; *n* = 3 mice; ns, P > 0.05; unpaired Student's *t* test. (C) ELISA shows that VEGF is up-regulated in the RPE/choroid of wild-type mice on D3 after laser injury in the CNV model (*n* = 4) compared with eyes before laser injury (*n* = 6); data are expressed as mean ± SEM; the asterisk indicates a significant increase in VEGF levels on D3 (***, P < 0.001; unpaired Student’s *t* test). (D) Pathological vascular leakage in *Nrp1^cyto/cyto^* mice and wild-type littermates. On D3 after laser injury in the CNV model, lesion size was assessed by fundus infrared (IR) imaging (left) before Evans Blue was injected intraperitoneally and dye leakage visualized 24 h later in eye sections counterstained with IB4 and DAPI; the Evans Blue single channel is shown in grayscale on the right hand side. Leakage into the retina at lesion level (as indicated by red) was quantified as the number of Evans Blue–positive pixels integrated for Evans Blue pixel intensity in mutants relative to littermate controls; mean ± SEM; *n* ≥ 8 mice each; *, P < 0.05 (unpaired Student’s *t* test). (E) Maximum intensity projections of confocal z-stacks through whole mount RPE/choroids from *Nrp1^cyto/cyto^* and wild-type littermates stained for IB4 on D14 after lasering in the CNV model. Quantification of lesion size (right) as number of IB4-positive pixels integrated for IB4-pixel intensity in mutants relative to littermate controls; mean ± SEM; *n* ≥ 5 eyes each; ns, not significant; P > 0.05 (unpaired Student’s *t* test). Bars: 25 µm (A); 1 mm (D, left); 200 µm (D, right); 200 µm (E).

To determine whether the NCD contributes to pathological vascular leak in the eye, we used a mouse model of choroidal neovascularisation (CNV) with pathological vascular changes similar to those observed in exudative AMD (Balaggan et al., 2006). In this model, three laser burns are applied to the retinal pigment epithelium (RPE) to rupture Bruch's membrane, causing inflammation and inducing VEGF-dependent CNV and vascular leakage (Balaggan et al., 2006). Consistent with a role for NRP1 in ocular vascular permeability, NRP1 is abundantly expressed in adult retinal and choroidal blood vessels (Fig. 7 A). However, and as observed in other organs, basal vascular permeability was unaffected in the retinal and choroidal vasculature of NCD-deficient mice (Fig. 7 B). We next injected Evans Blue systemically on day (D) 3 after lasering, when VEGF levels peak (Fig. 7 C), but CNV is only just beginning (Balaggan et al., 2006). We confirmed that lesion size was similar in *Nrp1^cyto/cyto^* and wild-type mice (Fig. 7 D) before injecting Evans Blue intraperitoneally. After 24 h, Evans Blue had extravasated around the laser lesion site at the level of the choroid and photoreceptors layers and into the inner retina (Fig. 7 D). Despite similar lesion size, dye leakage in the retina was significantly reduced in *Nrp1^cyto/cyto^* compared with wild-type mice (Fig. 7 D). In contrast, neoangiogenesis was unaffected in mutants, as histological analysis on D14 after laser injury showed similar neovascular lesion size in both genotypes (Fig. 7 E). Together, these findings suggest that the NCD does not promote pathological VEGF-induced angiogenesis, but selectively increases VEGF164-induced vascular permeability in adult neovascular eye disease.

## Discussion

NRP1 is a multifunctional protein essential in ECs for vascular development that is widely studied as a VEGF165 receptor (Lampropoulou and Ruhrberg, 2014; Raimondi et al., 2016). Whereas VEGF165-binding to NRP1 and complex formation with VEGFR2 were originally thought to drive angiogenesis, it was subsequently shown that VEGF164 binding to NRP1 makes only a small contribution to physiological angiogenesis in mice (Fantin et al., 2014; Gelfand et al., 2014). This conundrum was explained by the finding that NRP1 instead promotes postnatal angiogenesis through essential roles in extracellular matrix–induced actin cytoskeleton remodeling and TGFβ-modulated delta-notch signaling (Raimondi et al., 2014; Aspalter et al., 2015; Fantin et al., 2015). In contrast, the VEGF164-bound NRP1–VEGFR2 complex recruits GIPC1 to promote its trafficking into signaling endosomes, where it sustains pro-arteriogenic ERK1/2 signaling (Lanahan et al., 2013). However, it had not previously been examined whether VEGF164 binding to NRP1 or NCD-dependent GIPC1 recruitment contribute to VEGF164-induced vascular permeability, and it was not known whether NRP1 plays a role in SFK activation for VEGF164-induced vascular permeability. Here, we have shown that NRP1 binds VEGF164 to promote VEGF164/VEGFR2-induced vascular permeability, independently of GIPC1 (Fig. 3). These findings suggest that the NCD–GIPC1 interaction sustains VEGFR2 signaling to achieve high level ERK activation for arteriogenesis, whereas the NCD acts independently of GIPC1 to promote VEGF164-induced VEGFR2 signaling for SFK activation (Figs. 4, 5, and 6). Our findings in knock-in and knockout mouse models of NRP1 deficiency agree with earlier work in endothelial NRP1 mouse mutants, which had identified an essential role for NRP1 in VEGF164-induced vascular leakage (Acevedo et al., 2008). Although prior work did not determine why VEGFR2 is insufficient for vascular permeability induction, we now show that NRP1 is required as a VEGFR2 co-receptor to enable ABL-dependent SFK activation (Figs. 4, 5, and 6). Moreover, our finding that NRP1 regulates vascular permeability through ABL kinase activation also agrees and extends genetic studies implicating ABL kinases in VEGF164-induced vascular permeability (Aman et al., 2012; Chislock and Pendergast, 2013) by identifying the receptor complex that mediates ABL kinase activation.

A prior study used SU6656 to investigate the regulatory hierarchy of SRC and ABL kinase activation in ECs after VEGF164 stimulation and placed SRC upstream of ABL kinases (Chislock and Pendergast, 2013). However, this inhibitor targets both SRC and YES1, as well as VEGFR2, which resides at the top of this signaling cascade, and even targets ABL1, although to a smaller extent (remaining activity at 1 µM: SRC 31% and YES1 12% vs. VEGFR2 51% and ABL1 77%; Gao et al., 2013). Similarly, the SRC inhibitor PP2, which we have used here, is not selective for SRC, but has dual SFK/ABL kinase specificity (Tatton et al., 2003) and accordingly abrogated both VEGF165-induced SFK and ABL kinase activation (Fig. 5). Results obtained with these inhibitors therefore support the idea that the VEGFR2–ABL–SFK axis has a key role in VEGF164-induced permeability signaling, but they did not define the regulatory relationship of these kinases. In contrast, Imatinib does not block VEGFR2 activation (Fig. 5, F and G) and has high specificity for ABL kinases over SFKs (Deininger et al., 2005; Davis et al., 2011). Our results with Imatinib, when combined with those acquired with the VEGFR2 inhibitor PTK/ZK, therefore conclusively show that VEGFR2 is upstream of ABL kinases, which are upstream of SFKs (Fig. 5). These observations agree with those obtained with siRNA-mediated knockdown of ABL1 (Fig. 5, D and E). Hence, the finding that NRP1 cooperates with VEGFR2 to enable ABL-dependent SFK activation in an NCD-dependent fashion places several molecules previously reported to be essential for VEGF165-induced vascular permeability into a well-defined regulatory hierarchy (Fig. 5 I).

The observation that NRP1 forms a complex with ABL1 in ECs independently of VEGF165 stimulation (Raimondi et al., 2014) raises the possibility that NRP1 helps deliver ABL1 to VEGFR2, once VEGF165 has induced complex formation between NRP1 and VEGFR2. In this manner, NRP1-bound ABL1 would be able to phosphorylate SFKs that are recruited to VEGFR2 via SH2D2A, the intracellular adaptor protein that binds the phosphorylated Y951 residue of VEGFR2 that is required for VEGF164-induced vascular permeability (Sun et al., 2012; Li et al., 2016). Supporting this idea, SFK activation by ABL kinases would require spatial proximity of both types of proteins, because ABL kinases depend on the interaction with their substrates to overcome intramolecular autoinhibition (Wang, 2014), and such proximity would be instilled when VEGF165 tethers the VEGFR2–SH2D2A–SFK and NRP1–ABL1 complexes to each other by forming a bridge between its two receptors. This model of higher order complex formation between several signaling components in the VEGF pathway is consistent with the strong reduction in pSFK levels after NRP1 or ABL1 knockdown (Fig. 5), as well as our genetic studies, which showed that endothelial NRP1 and VEGF164-binding to NRP1 are both required for a robust permeability response (Figs. 2 and 3). Moreover, an important role of the NCD in this pathway agrees with prior observations that the NCD enhances complex formation of NRP1 and VEGFR2 in VEGF165-stimulated ECs (Prahst et al., 2008) and promotes ABL1 function in tumor cells (Yaqoob et al., 2012).

Prior work in arterial ECs showed that GIPC1 interacts with the NCD to sort the VEGF164-activated VEGFR2 receptor complex into cellular compartments devoid of phosphatases that would otherwise dephosphorylate the VEGFR2 residue Y1173 (Lanahan et al., 2013). In contrast, our observation that GIPC1 is dispensable for the VEGF164-induced permeability signaling (Fig. 3), which depends on Y949 phosphorylation of VEGFR2 (Sun et al., 2012; Li et al., 2016), raises the possibility that NCD-mediated intracellular VEGFR2 trafficking, even though important to maintain the VEGFR2 (Y1173)–ERK1/2 axis, is not important for activation of the VEGFR2 (Y949)–ABL–SFK axis.

Downstream of the VEGF165-induced signal transduction cascade via VEGFR2, different cellular mechanisms have been implicated in the induction of vascular leakage. For example, VEGF165 has variably been suggested to stimulate the formation of vesiculo-vacuolar organelles for transcellular leakage (Dvorak et al., 1996; Bates and Harper, 2002) or disrupt adherens junctions between adjacent ECs to increase paracellular leakage (Dejana et al., 2008). For paracellular leakage, SFK-mediated FAK activation regulates adherens junction dynamics by promoting the dissociation of CTNNB1 (β-catenin) from CDH5 (Chen et al., 2012). Whether the NRP1 pathway identified here controls VEGF165-induced permeability predominantly by promoting adherens junction breakdown and/or a transcellular transport remains to be evaluated.

The residual vascular permeability observed in the Miles assay with mice lacking endothelial NRP1, VEGF164-binding to NRP1, or the NCD may be explained by a low level of VEGFR2-mediated permeability signaling, independently of NRP1. A possibility to explain this observation may be that the NRP1-independent pathway utilizes ABL2 for SFK activation, because ABL2 can be activated by VEGF164 (Aman et al., 2012; Chislock and Pendergast, 2013). Supporting the idea that ABL2 can help convey VEGF164-induced VEGFR2-mediated permeability signaling, we show here that ABL1 knockdown or NRP1 does not completely abolish pCRKL induction, whereas the pharmacological VEGFR2 blockade with PTK/ZK or dual ABL1/ABL2 blockade with Imatinib abrogated pCRKL and pSFK induction in response to VEGF164 in vitro. Agreeing with a model in which VEGFR2 is upstream of both ABL1 and ABL2 activation, it has been shown that ABL2 partially compensates for ABL1 in VEGF164-induced vascular leakage in the Miles assay (Chislock and Pendergast, 2013). It remains to be investigated how ABL2, which remains active in VEGF164-stimulated ECs after NRP1 knockdown, but not VEGFR2 inhibition, might be recruited to VEGFR2 during the permeability response. Nevertheless, our finding that VEGFR2 is indispensable for VEGF164-induced SFK activation and vascular leakage agrees with prior permeability studies using inhibitors that have VEGFR2 as one of their targets (Murohara et al., 1998), a recent study using function-blocking antibodies for VEGFR2 (Hudson et al., 2014) and mouse mutants lacking VEGFR2 Y951 phosphorylation (Li et al., 2016). On the other hand, NRP1 can convey C-end-Rule peptide-mediated leakage independently of VEGFR2 activation (Roth et al., 2016), although the precise downstream mechanism remains to be elucidated.

It is presently unclear why NRP1 function-blocking antibodies have yielded variable results in disrupting VEGF164-induced vascular permeability, with some studies suggesting a strong inhibitory effect (Becker et al., 2005; Teesalu et al., 2009), others observing permeability induction (Roth et al., 2016) and others observing no effect (Pan et al., 2007; Acevedo et al., 2008). One possibility is that the delivery method or epitopes specificity affect an antibody's ability to modulate NRP1 function. Importantly, these limitations do not apply to genetic mouse models such as those used in the present study, because tissue-specific NRP1 expression or NRP1 domain functions have been targeted in a clearly defined, uniform, and easily validated manner. Thus, our analyses of intradermal and ocular vascular leakage, as well as SFK activation in genetic mouse models conclusively show that NRP1 is required for vascular permeability signaling by binding VEGF164 and relaying signals through its NCD to promote SFK activation. Interestingly, the alternative NRP1 ligand SEMA3A also induces acute vascular permeability via NRP1, but it does not rely on SFK activation (Acevedo et al., 2008). Accordingly, the VEGF164 and SEMA3A permeability pathways have been proposed to diverge, despite their shared NRP1 dependence. In agreement, we have found that the NCD, even though required for VEGF164-induced SFK activation and vascular leakage, is dispensable for SEMA3A-induced vascular leakage (Fig. 3).

Excessive vascular permeability impairs sight in eye diseases such as exudative age-related macular degeneration (AMD), diabetic retinopathy, and retinal vascular occlusion (Campochiaro, 2015). Although various processes increase vascular permeability, pathological leakage in the eye most strongly correlates with raised intraocular VEGF (Vinores et al., 1997; Rosenfeld et al., 2006; Campochiaro, 2015). Accordingly, edema in AMD can be significantly reduced with anti-VEGF therapies targeting all VEGF isoforms, including an anti-VEGF antibody or Fab fragment and a VEGF trap (Campochiaro, 2015). Although effective against edema, studies in mouse models suggest that global VEGF blockade might adversely affect long-term eye health. Thus, reducing VEGF levels in the mouse eye compromises the maintenance of the choroidal vasculature that is essential for photoreceptor health (Saint-Geniez et al., 2009). Moreover, inhibiting all VEGF signaling impairs the survival of retinal neurons in a mouse model of retinal ischemia, but VEGF120 is capable of restoring neuroprotection via VEGFR2 (Nishijima et al., 2007). An aptamer that selectively targets VEGF165 may provide an alternative to treat ocular edema when inhibiting all VEGF isoforms is not appropriate (Ng et al., 2006), although clinical data comparing long term eye safety of this therapeutic and the more commonly used pan-VEGF inhibitors is not presently available.

Our findings raise the possibility that NRP1-based therapeutics might provide an alternative approach to treating vascular leakage in eye disease when anti-VEGF treatment is not suitable or effective. One possible strategy might be to inhibit VEGF165-mediated permeability signaling via the NCD or its interactors. This idea is based on our finding that NCD-deficient mice have reduced vascular leakage in a mouse model of CNV that has several hallmarks of neovascularisation and vascular hyperpermeability in AMD (Fig. 7). As NCD loss does not impair physiological angiogenesis (Fantin et al., 2011) and does not compromise the survival of VEGF164-dependent neurons, at least outside the central nervous system (Cariboni et al., 2011), targeting NCD-dependent permeability signaling may be particularly useful for conditions in which there is a need for VEGF120-mediated cytoprotection or the formation of new vasculature, such as in the ischemic eye. An alternative strategy to prevent ocular vessel leakage may involve targeting both VEGF164 and SEMA3A signaling via NRP1, in particular in conditions where SEMA3A exacerbates VEGF-induced leakage, as has been proposed for the early phase of diabetic macular edema (Cerani et al., 2013). As SEMA3A induces vascular leakage in the Miles assay via NRP1, but independently of the NCD (Fig. 3) or SFKs (Acevedo et al., 2008), reducing VEGF165- and SEMA3A-induced leakage would require blockade of both ligand-binding domains in NRP1 or inhibiting a common downstream target. Future work is therefore warranted to investigate whether targeting NRP1-mediated signaling is effective in treating edema in the eye or other organs to offer a therapeutic approach that preserves the valuable vaso- and neuroprotective functions of the VEGF isoforms that do not bind NRP1.

## Materials and methods

### Study approval

Animal work was performed following UK Home Office and institutional Animal Welfare and Ethical Review Body (AWERB) guidelines.

### Mouse strains

To create mice with an endothelial-specific NRP1 or VEGFR2 deletion, we mated floxed conditional *Nrp1*-null (*Nrp1^fl/fl^*; Gu et al., 2003) and *Vegfr2*-null mice (*Vegfr2^fl/fl^*) to mice carrying the endothelial-specific *Cdh5-CreERT2* (Zarkada et al., 2015). To induce gene deletion, sex-matched Cre-positive and Cre-negative young adult littermates were injected with 0.5 mg tamoxifen (Sigma-Aldrich) in peanut oil twice weekly for 2 wk up until 2 d before performing Miles assays (see following section). Mice carrying one *Nrp1*-null allele (*Nrp1^+/−^*), mice with two *Nrp1* alleles deficient in VEGF binding (*Nrp1^Y297A/Y297A^*), NCD-deficient mice (*Nrp1^cyto/cyto^*), and *Gipc1*-null mice (*Gipc1^−/−^*) have been described previously (Kawasaki et al., 1999; Chittenden et al., 2006; Fantin et al., 2011, 2014). *Nrp1^D320K/D320K^* mice carrying a previously described mutation that abrogates VEGF164 binding to NRP1 (Gelfand et al., 2014) were generated with CRISPR/CAS9 technology by the Gene Targeting and Transgenic Facility of the University of Connecticut Health Centre. All mice were maintained on a C57BL/6J background (The Jackson Laboratory).

### Miles assay

Both flanks of adult anaesthetized mice were shaven. The next day, 100 µl of 1% Evans Blue (Sigma-Aldrich) in sterile saline (wt/vol) was injected intravenously through the lateral tail vein. In some experiments, mice received an intraperitoneal injection of pyrilamine maleate (4 mg/kg body weight in 0.9% saline; Sigma-Aldrich) before Evans Blue injection to inhibit release of endogenous histamine. 30 min after Evans Blue injection, 20 µl of PBS or PBS containing 50 ng VEGF164 (PeproTech), 300 ng SEMA3A (R&D Systems), or 50 ng histamine (Sigma-Aldrich) were injected intradermally each at three sites into the flank skin of anaesthetized mice. After 20 min, mice were culled, the skin was separated from the underlying muscle, and the tissue surrounding the injection sites excised (circled in Fig. 1 A) and dried overnight at 55°C. Evans Blue was extracted by incubation in formamide at 55°C overnight and quantified by spectrophotometry at 620 nm after subtraction of background absorbance at 740 nm. Data from the three sites injected with the same agent (ligand or PBS) were averaged and expressed as fold change relative to PBS control per each mouse. In some experiments, the inner side of the skin was imaged on an MZ16 stereomicroscope (Leica) equipped with a Micropublisher camera (Perkin-Elmer). In other experiments, a sample of liver or skin tissue was retained for immunoblotting.

### Baseline permeability assay

100 µl 2% Evans Blue in PBS (wt/vol) was injected intravenously through the lateral tail vein of adult mice and left to circulate for 24 h. A blood sample was taken from the heart of the anesthetized mice followed by transcardial perfusion with 1% formaldehyde in PBS containing 0.05 M sodium citrate, pH 4.2, prewarmed to 37°C, to clear circulating dye before organ collection, dry weight measurement, dye extraction, and quantification. Absorbance values were normalized to tissue weight and Evans Blue blood levels, except for the retina and RPE/choroid/sclera complex, in which case the value per eye tissue was normalized to Evans Blue blood levels.

### Cell culture

HDMECs were cultured in MV2 media with supplements (Promocell). MBECs were isolated from mice between 1 and 3 wk of age and cultured on tissue culture plates coated with 20 μg/ml FN in EGM2 media (Lonza) without passaging; 4 µg/ml puromycin (Sigma-Aldrich) was included in the media for 2 d to eliminate contaminating cell types. MLECs were isolated from mice between 1 and 2 mo of age by magnetic-activated cell sorting (MACS) with PECAM1 and ICAM2 antibodies (BD). MLECs were cultured on tissue culture plates coated with 10 µg/ml FN in DMEM-GlutaMAX supplemented with 20% FBS, nonessential amino acids (Life Technologies), and ECGS (Sigma-Aldrich). Cells were stimulated with 50 ng/ml VEGF165 (PeproTech; for HDMEC) or VEGF164 (for MLECs or MBECs) for the indicated times. In some experiments, HDMECs were incubated with inhibitors dissolved in DMSO or the same concentration of DMSO 30 min before VEGF165 stimulation. We used the following inhibitors: 10 µM Imatinib (Cambridge Bioscience), 10 µM PP2 (Sigma-Aldrich), and 0.1 µM PTK/ZK (Vatalanib; Selleckchem).

### Immunofluorescence

Adult anesthetized mice were transcardially perfused with PBS, and then 4% formaldehyde in PBS. Eye and ear tissue samples were whole mount immunostained as described previously (Fantin et al., 2015) using the following primary antibodies: rat anti–mouse PECAM1 (BD), FITC-conjugated mouse anti-SMA (Sigma-Aldrich) or rabbit anti-CDH5 (from P. Turowski, University College London, London, England) and goat anti–rat NRP1 (R&D Systems), previously shown to recognize mouse NRP1 (Fantin et al., 2010), followed by Alexa Fluor 488–, 594–, or 647–conjugated donkey anti–rabbit, anti–rat, or anti–goat secondary antibodies (Jackson ImmunoResearch Laboratories). Confocal z-stacks were acquired with C-Apochromat 10× 0.45 NA and 40× 1.2 NA water objectives a LSM710 laser scanning confocal microscopes (ZEISS). HDMEC and MLEC were fixed in 4% formaldehyde in PBS for 15 min. To detect NRP1 in HDMEC, we used mouse anti–human NRP1 (R&D Systems). In some experiments, cultured cells were incubated with the anti-NRP1 antibody for 5 min before fixation (nonpermeabilizing conditions). We also used goat anti–human CDH5 (Santa Cruz Biotechnology, Inc.) and rabbit anti–human pSRC (Cell Signaling Technology). To visualize primary antibodies, we used Alexa Fluor 488– or 647–conjugated donkey anti–rabbit or anti–goat secondary antibodies (Jackson ImmunoResearch Laboratories). Cells were counterstained with DAPI (Sigma-Aldrich) and imaged with a Plan-Apochromat 63× 1.4 NA oil objective on a LSM700 confocal microscope (ZEISS). Images were processed with Photoshop CS4 (Adobe).

### Immunoblotting

Anesthetized mice were injected intradermally with 10 µl PBS in one ear and with 10 µl PBS containing 50 ng VEGF164 in the other ear. After 20 min, mice were culled, and the ear tissue surrounding the injection site was used for immunoblotting. These ear biopsies, as well as dermis and liver tissue from tamoxifen-treated mice and their littermate controls, was homogenized with a pestle, lysed in Laemmli sample buffer, and sonicated. Cells were lysed in 50 mM Tris-HCl, pH 7.4, 5 mM EDTA, 0.1% NP-40, 250 mM NaCl in the presence of protease inhibitor cocktail 2 and phosphatase inhibitor cocktail (Sigma-Aldrich). Denatured proteins were separated by SDS-PAGE and transferred to nitrocellulose membrane (Whatman) for immunoblotting with the following primary antibodies: rabbit anti–human pVEGFR2-Y1175, rabbit anti–human pVEGFR2-Y951, rabbit anti–human VEGFR2, rabbit anti-SRC, rabbit anti–human pSRC-Y4169, rabbit anti–human pERK1/2-T202/Y204, rabbit anti–rat ERK1/2, rabbit anti–human pCRKL-Y207, rabbit anti–mouse AKT, rabbit anti-NRP1 (Cell Signaling Technology), rabbit anti–human GAPDH (Abcam), rabbit anti–human CRKL, and goat anti–human CDH5 (Santa Cruz Biotechnology, Inc.).

### CNV assay

CNV was induced with a diode laser as previously described (Balaggan et al., 2006). In brief, three laser lesions per eye were delivered at three disc diameters away from the optic nerve head into adult anesthetized mice using a slit-lamp-mounted diode laser system (Keeler). On D3 post-lasering, fundus infrared imaging was performed with a scanning laser ophthalmoscope (Heidelberg Spectralis) to ensure that lesion size was similar in mutants and controls before 250 µl Evans Blue (1% in saline, wt/vol) were injected intraperitoneally. Eyes were harvested 24 h later, fixed in 4% formaldehyde, cryosectioned and stained with biotinylated isolectin B4 (IB4; Sigma-Aldrich) followed by Alexa Fluor 647–conjugated streptavidin (Thermo Fisher Scientific) and counterstaining with DAPI. In some experiments, the retinal pigment epithelium (RPE)/choroid complex was dissected on D14 and stained whole mount with IB4. Samples were imaged as described above. Retinal vascular leakage was measured as density of Evans Blue–positive pixels integrated for Evans Blue pixel intensity in maximum projections of confocal z-stacks through each lesion. The size of each CNV lesion was quantified in maximum projections of confocal z-stacks as density of IB4-positive pixels integrated for IB4-pixel intensity. D14 lesion sizes and D3 lesion leakage were averaged for each mouse and expressed as fold change in mutants relative to littermate controls.

### Statistics

To determine if two datasets were significantly different, we calculated the P-value with Excel 12.2.6 (Microsoft Office) in two-tailed paired or unpaired Student’s *t* tests, as described in the figure legends; P < 0.05 was considered significant. Error bars represent the standard error of the mean, unless stated differently in the text.
